# Transcriptional Organization of the *Salmonella* Typhimurium Phage P22 *pid* ORFan Locus

**DOI:** 10.3390/ijms23031253

**Published:** 2022-01-23

**Authors:** Sanne Wolput, Angela Makumi, Laura Wicke, Leonard E. Bäcker, William Cenens, Yves Briers, Nicolas A. Wenner, Siân V. Owen, Jay C. D. Hinton, Rob Lavigne, Abram Aertsen

**Affiliations:** 1 Laboratory of Food Microbiology, Department of Microbial and Molecular Systems, KU Leuven, 3000 Leuven, Belgium; sanne.wolput@kuleuven.be (S.W.); A.Makumi@cgiar.org (A.M.); leon.backer@kuleuven.be (L.E.B.); w.cenens@gmail.com (W.C.); 2 Laboratory of Gene Technology, Department of Biosystems, KU Leuven, 3000 Leuven, Belgium; laura.wicke@kuleuven.be (L.W.); rob.lavigne@kuleuven.be (R.L.); 3 Laboratory of Applied Biotechnology, Department of Biotechnology, Ghent University, 9000 Ghent, Belgium; Yves.Briers@UGent.be; 4 Institute of Infection, Veterinary and Ecological Sciences, University of Liverpool, Liverpool CH64 7TE, UK; nicolas.wenner@unibas.ch (N.A.W.); sian@hms.harvard.edu (S.V.O.); Jay.Hinton@liverpool.ac.uk (J.C.D.H.)

**Keywords:** phage–host interactions, *Salmonella* Typhimurium, phage P22, *pid* ORFan locus, *dgoRKAT* operon

## Abstract

Many phage genes lack sequence similarity to any other open reading frame (ORF) in current databases. These enigmatic ORFan genes can have a tremendous impact on phage propagation and host interactions but often remain experimentally unexplored. We previously revealed a novel interaction between phage P22 and its *Salmonella* Typhimurium host, instigated by the ORFan gene *pid* (for phage P22 encoded instigator of *dgo* expression) and resulting in derepression of the host *dgoRKAT* operon. The *pid* gene is highly expressed in phage carrier cells that harbor a polarly located P22 episome that segregates asymmetrically among daughter cells. Here, we discovered that the *pid* locus is fitted with a weak promoter, has an exceptionally long 5′ untranslated region that is instructive for a secondary *pid* mRNA species, and has a 3′ Rho-independent termination loop that is responsible for stability of the *pid* transcript.

## 1. Introduction

With an estimated presence of around 10^31^ virions, viruses of bacteria (i.e., bacteriophages or phages) are the most ubiquitous entities in our biosphere and have an unrivalled impact on the ecology and evolution of their hosts [1,2]. Furthermore, the many different phage species that exist constitute a tremendous sequence space that we have only just begun to explore [3]. Indeed, while sequencing efforts are massively sampling this space, it is clear that many phage genes lack homologs in the currently existing databases and have not been investigated experimentally [4,5,6]. However, these unknown phage genes (also referred to as phage ORFans) can profoundly impact phage propagation [7,8] and host interactions [9,10]. 

As such, the existence of many phage ORFan genes underscores that the full complexity of phage–host associations and interactions has yet to be established, even in well-studied model systems such as the temperate lambdoid phage P22 and its *Salmonella* Typhimurium host. Indeed, we have previously revealed the existence of a cryptic carrier state association of P22 that deviates from its strictly lytic or lysogenic proliferation [11]. We found that during the path towards lysogenisation of its host, P22 is able to form a polarly-located episome that persistently becomes segregated asymmetrically between daughter cells. In carrier cells inheriting this P22 episome, a P22 ORFan gene termed *pid* (for phage P22 encoded instigator of *dgo* expression [12]) is highly expressed. The corresponding Pid protein derepresses the host *dgoRKAT* operon, an operon that drives D-galactonate metabolism [12], although the impact of this Pid/*dgo* interaction and the regulation of the *pid* locus remain elusive. 

The presence of the *pid* locus remained unnoticed for decades, until its identification as a functional gene, located between the *orf25* and *orf80* of P22 [12]. Although found in the late region of the P22 genome, the orientation of *pid* opposes the main late promoter. In this study, we report the findings of a genetic screen designed to reveal key regulatory functions of the P22 *pid* locus. 

## 2. Results

### 2.1. Genetic Screen for Functional Mutations in the P22 pid Locus 

**Figure 1:**
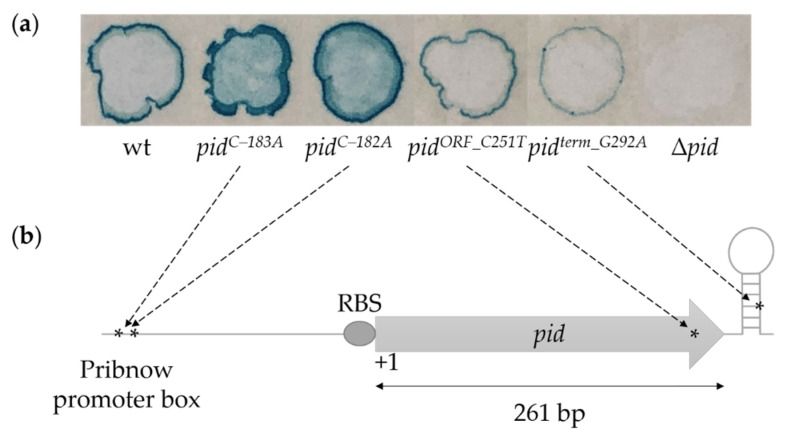
DES-mutagenesis screen revealed functional mutations in the P22 *pid* locus. Selected DES-mutagenized P22 clones plaqued on a lawn of the LT2K7 indicator (LT2 *dgoT*::Mu*d*K) on LB X-Gal in which blue coloration reports on LacZ activity (panel (**a**)) and the corresponding mutation in the *pid* locus (panel (**b**)). Numbering of the mutations (indicated as *) is relative to the *pid* start codon.

To functionally characterize the P22 *pid* locus, we set up a genetic screen in which a DES-mutagenized P22 population was plaqued on LB X-Gal on the LT2K7 indicator strain (i.e., LT2 *dgoT::*Mu*d*K; [12]) that reports on derepression of the *dgo* operon by producing the LacZ reporter protein. We chose plaques with an altered degree of blue color resulting from LacZ activity on the X-Gal and sequenced the *pid* locus of the corresponding phage. We excluded clear plaques from this screen since previous research revealed that obligate lytic variants of P22 do not enter the carrier state and do not express Pid, and therefore do not support the Pid/*dgo* interaction [12]. Our screen revealed (i) two clones (P22-*pid^C–183A^* and P22-*pid^C–182A^*) that had more intense blue coloration than wild-type P22 (i.e., P22wt) and that carry mutations far upstream of the *pid* open reading frame (ORF), (ii) one blue-attenuated clone (P22-*pid^ORF_C251T^*) carrying a mutation within the *pid* ORF, and (iii) one blue-attenuated clone (P22-*pid^term_G292A^*) carrying a mutation downstream of the *pid* ORF (Figure 1).

Of the two mutants attenuated in the Pid/*dgo* interaction, one contained a C-to-T mutation located 251 bp downstream of the *pid* start codon, leading to a T84M amino acid substitution in the C-terminal part of the Pid protein. However, since the structure–function relationship of Pid currently remains elusive, it was unclear as to how this amino acid change would compromise the Pid protein or the Pid/*dgo* interaction. The other mutant harbored a G-to-A mutation in the stem-loop structure downstream of the *pid* ORF (292 bp downstream of the *pid* start codon). This stem-loop (hairpin) structure is characterized by a G+C-rich stem followed by a stretch of poly(T) on the sense strand, likely corresponding to a Rho-independent termination site [13]. Such sites play an important role in the termination mechanism by regulating the release of the transcript and RNA polymerase from the DNA template strand. The structure and dimension of such factor-independent termination sites can vary, and can influence transcript termination [14]. The G-to-A mutation in the G+C-rich stem structure of *pid* might disrupt stem-loop formation and impact upon termination functionality and mRNA stability. 

### 2.2. Characterization of the P22 pid Promoter 

Since the above-mentioned mutations within and downstream of the *pid* ORF are likely loss-of-function mutations that simply compromise the Pid/*dgo* interaction, subsequent focus was placed on the upstream mutations that appeared to boost the interaction, i.e., P22-*pid^C–183A^* (with a C-to-A mutation, located 183 bp upstream of the *pid* start codon) and P22-*pid^C–182A^* (with a C-to-A mutation, located 182 bp upstream of the *pid* start codon). Since both mutations were next to each other but far upstream of the *pid* start codon, the surrounding genetic context was scrutinized and predicted to be a promoter (97.1% probability score via the *PhagePromoter* prediction tool integrated in the Galaxy framework, [15]). Moreover, a possibly eroded Pribnow promoter box (i.e., 5′-AATCCT-3′ vs. the canonical 5′-TATAAT-3′) was identified 181–186 bp upstream of the start codon where both the C-to-A mutations were situated (*p*-value of 0.0014 via the *SAPPHIRE* 1 Promoter prediction tool, [16]).

**Figure 2:**
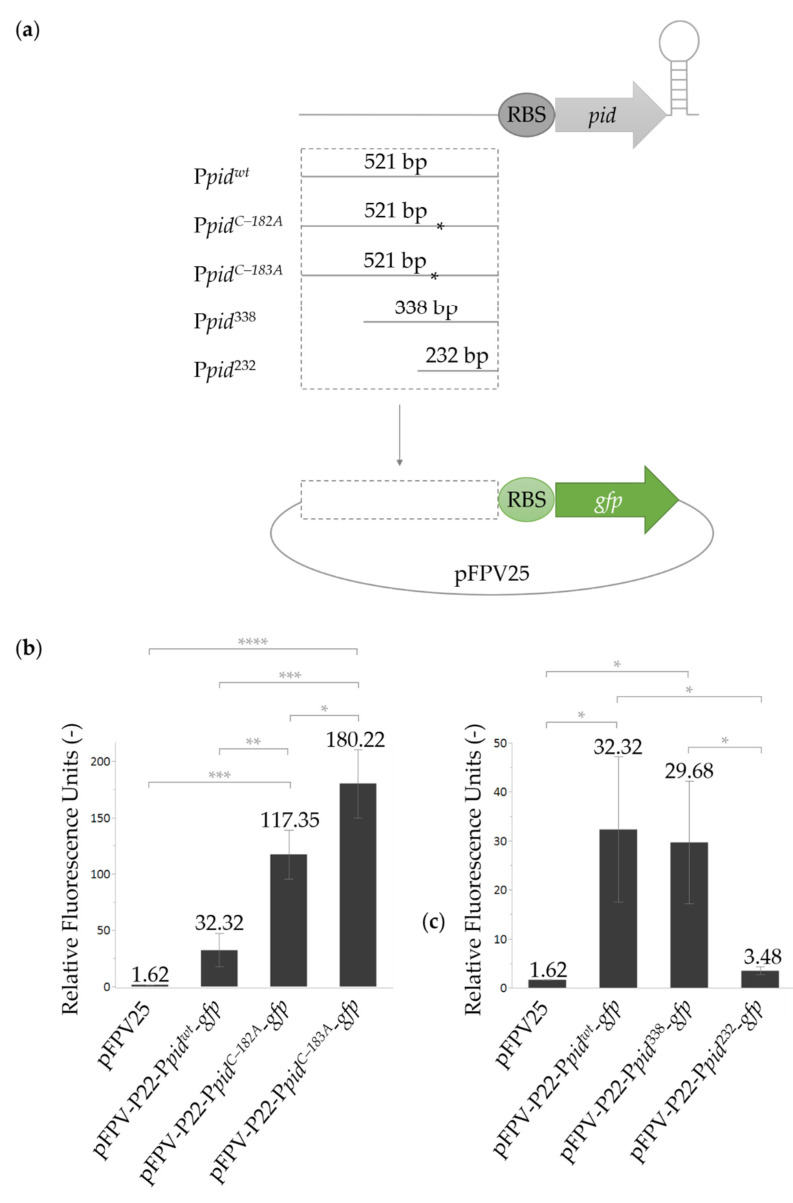
Assessment of the activity of the wild-type and mutant versions of the *pid* promoter. (**a**) Schematic of the different *pid* promoter variants constructed upstream of the *gfp* gene in the pFPV25 promoter probe plasmid. (**b**,**c**) GFP fluorescence stemming from AB-grown stationary phase cultures of LT2 equipped with the indicated reporter plasmids (panel (**b**): promoter mutations; panel (**c**): promoter truncations; as depicted in panel (**a**)). Please note that pFPV25 and pFPV-P22-P*_pid_^wt^-gfp* are the same samples in panel (**b**) and (**c**). The mean fluorescence intensity relative to the OD_600_ value is represented in bars, and the mean value and the standard deviation of three biological repeats are shown. The empty plasmid (pFPV25) was included as a negative control. Significant differences (Tukey HSD test) are indicated by * (*p* ≤ 0.05), ** (*p* ≤ 0.01), *** (*p* ≤ 0.001), and **** (*p* ≤ 0.0001).

To experimentally investigate the functionality of this putative promoter region, and of the C–182A and C–183A mutations, the fragment from 533 to 12 bp upstream of the *pid* start codon from P22wt, P22-*pid^C–182A^*, and P22-*pid^C–183A^* was cloned upstream of the *gfp* gene in the pFPV25 promoter-probe vector [17] and assayed for GFP fluorescence production (Figure 2a). It was clear that the upstream region contained a basal promoter activity, which was boosted four- or sixfold by the C–182A or C–183A mutation, respectively (Figure 2b). These mutations change the region into 5′-AATCAT-3′ or 5′-AATACT-3′, respectively, and likely improve the functionality of the Pribnow promoter box by more closely resembling the optimal prokaryotic consensus sequence (i.e., 5′-TATAAT-3′). Comparable studies on other genes have shown that mutations that make the Pribnow box more similar to the consensus sequence cause increased transcription of the downstream gene [17,18,19]. When the wild-type fragment cloned upstream of the *gfp* reporter gene was shortened from its 5′ end (cf. P*_pid_*^338^ and P*_pid_*^232^; Figure 2a), promoter activity was lost when the 533 to 245 bp fragment upstream of the *pid* start codon was removed (i.e., P*_pid_*^232^; Figure 2c), suggesting no other promoters were present within the 244 bp fragment immediately upstream of the *pid* ORF. 

**Figure 3:**
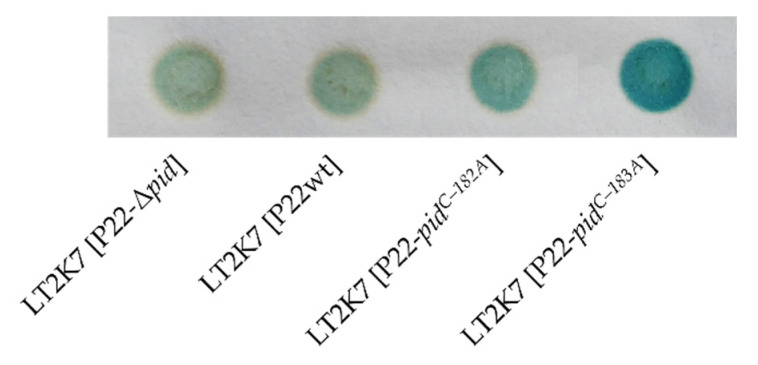
Pid/*dgo* interaction is absent during lysogeny. Spots of indicated LT2K7 (i.e., LT2 *dgoT::*Mu*d*K) lysogens grown on LB X-Gal in which blue coloration reports on LacZ activity, and thus *dgo* derepression.

Finally, the plasmid data in Figure 2 also suggest that the wild-type *pid* promoter (P*_pid_^wt^*) is active (or at least shows constitutive leaky expression), even in the absence of P22-borne regulatory factors. Nevertheless, the leaky expression of *pid* from a P22wt lysogen (in the LT2K7 reporter) was insufficient to mount the Pid/*dgo* interaction (Figure 3). In contrast, a P22-*pid^C–183A^* lysogen with upregulated P*_pid_* activity constitutively derepressed the *dgo* operon (Figure 3).

### 2.3. Characterization of the P22 pid Transcript

**Figure 4:**
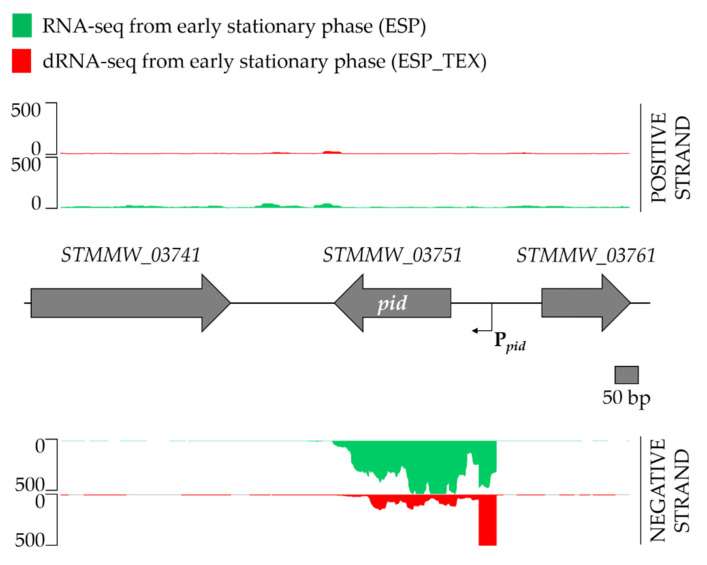
Identification of the transcription start site of the *pid* locus in prophage BTP1. RNA-sequencing reads mapped to the positive and negative strand are shown in the upper and lower panels, respectively. The scale is 0–500 normalized reads. Normalized RNA-seq data from early stationary phase are shown in green, and differential (d)RNA-seq data from early stationary phase are shown in red. dRNA-seq data are derived from RNA treated with terminator exonuclease (TEX) to enrich for transcripts carrying a 5′ triphosphate (primary transcripts), enabling more precise identification of transcription start sites. The TSS of *pid* (indicated with an arrow) was located 174 bp upstream of the start codon of the *pid* coding sequence, at nucleotide position 389,658 on the D23580 chromosome. RNA-seq and dRNA-seq data were reinterpreted from Hammarlöf et al. (2018) [21].

Because the P*_pid_* promoter appeared to be far upstream of the start codon, the *pid* transcript was predicted to have an extensive 5′ untranslated region (5′UTR). Analysis of recent RNA-seq data from the *Salmonella* Typhimurium D23580 strain [6,20,21,22], which is naturally lysogenized with the BTP1 prophage that carries a *pid* locus identical to that of P22, revealed that the transcriptional start site (TSS) of P*_pid_* was indeed located 174 bp upstream of the *pid* start codon. This TSS location further confirms that the mutations identified in the DES-mutagenesis screen were located in the Pribnow box of the promoter region. Specifically, the C–182A and C–183A mutations modulated the –8 and –9 regions of the P*_pid_* promoter (Figure 4). These data suggest that the TSS of the *pid* gene is located at nucleotide 40,880 on the genome of the P22 phage (NCBI Reference Sequence NC_002371.2).

**Figure 5:**
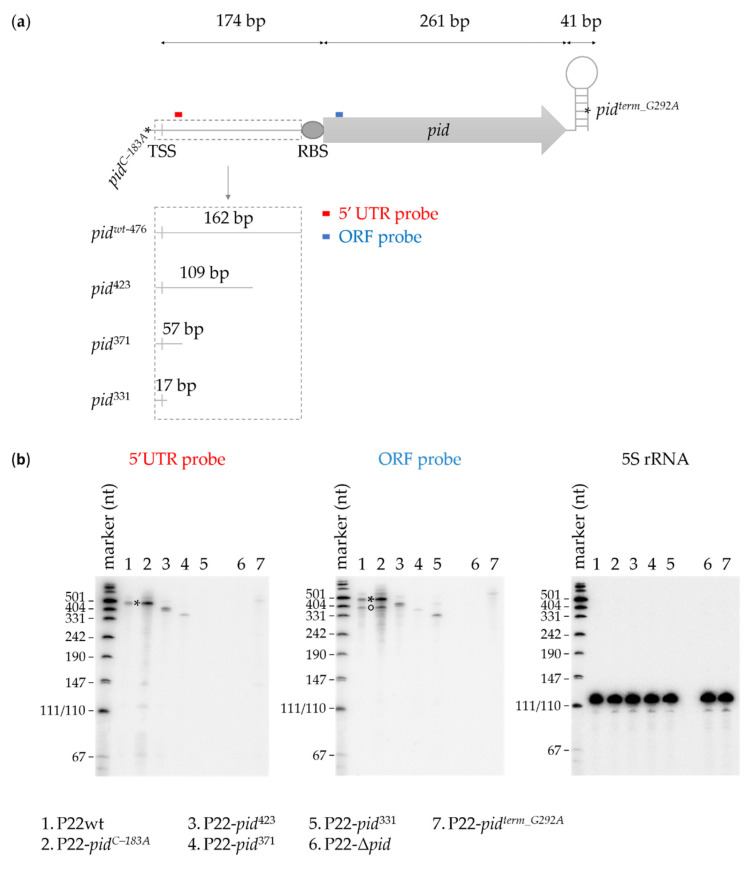
Visualization of the wild-type and mutant *pid* transcripts. (**a**) Scheme depicting the *pid* locus of P22wt and its indicated mutants and including the position of the ssDNA Northern blotting probes targeting either the 5′UTR region or the ORF region of the transcript. (**b**) Representative images of the Northern blots of the *pid* transcript expressed by P22wt and the indicated P22 mutants during high MOI infection of LT2. Northern blots were labelled with either the 5′UTR (left panel) or ORF (middle panel) probe, and infection with P22-Δ*pid* was included as a negative control. The 5S rRNA loading controls are shown in the right panel. The F- and S-bands of lanes 1–2 are marked by * and °, respectively.

The length of the *pid* transcript of P22 was examined in more detail via Northern blotting. To ensure *pid* expression, we exposed LT2 populations to a high MOI infection with wild-type or mutant P22 in order to favor lysogenisation and phage carrier state dynamics. After RNA extraction of these infected populations, Northern blotting involved either a ssDNA probe targeting the 5′UTR region of the *pid* mRNA or a ssDNA probe targeting the ORF region (Figure 5a). Following P22wt infection, the 5′UTR probe revealed a transcript corresponding to the expected full-length of 476 bp spanning from the TSS to the Rho-independent termination site (referred to as F-band) (Figure 5b, lane 1). Following infection with P22-*pid^term-G292A^* (carrying the G-to-A terminator mutation 292 bp downstream of the *pid* start codon), the 5′UTR probe only revealed a vague F-band (Figure 5b, lane 7), supporting our earlier hypothesis (Figure 1) that Rho-independent termination was important for *pid* mRNA stability. As expected, no *pid* transcript was observed following a negative control infection with P22-Δ*pid*, in which the *pid* ORFan had been removed (from the promoter region, 240 bp upstream of the start codon, to the Rho-independent termination site) (Figure 5b, lane 6).

Interestingly, following P22wt infection, the ORF probe not only revealed the expected F-band, but also a slightly smaller fragment or transcript of between 350 and 400 bp (referred to as smaller or S-band) (Figure 5b, lane 1). To further investigate whether the 5′UTR was the cause of this S-band, we made truncations within the 5′UTR of the *pid* locus in P22, from the 3′ end, while leaving the RBS of *pid* intact. The resulting mutant phages then contained the *pid* promoter region extending into either 109 bp (P22-*pid*^423^), 57 bp (P22-*pid*^371^), or 17 bp (P22-*pid*^331^) of the 5′UTR region, followed by the *pid* RBS and ORF (Figure 5a). Northern blotting of the RNA extracted from high MOI infections with these truncated mutants yielded correspondingly shortened F-bands with both the 5′UTR or ORF probe (note that the annealing position of the 5′UTR probe has been removed in P22-*pid*^331^) (Figure 5b, lanes 3–5). However, no parallel shortened S-bands could be detected with the ORF probe in these samples, suggesting that the 5′UTR region 110–162 bp downstream of the transcriptional start site is responsible for this S-band.

This might in turn indicate that the 5′UTR region contains either secondary promoter elements or a processing site, leading to a smaller (S-band) transcript or fragment next to the full length (F-band) transcript. However, our promoter-probe studies detailed above (Figure 2) did not indicate additional promoters to be present closer to the *pid* ORF. Moreover, boosted *pid* expression via infection with P22-*pid^C–183A^* (carrying the upregulating C-to-A mutation at –9 from the TSS) not only revealed the F- and S-bands with the ORF probe, but also a number of shorter fragments that might hint towards processing of the F-band (Figure 5b, lane 2).

## 3. Discussion

Our investigation of the transcriptional organization of the P22 *pid* locus showed that the promoter was slightly leaky, and that expression of the *pid* transcript was mainly attenuated because of an eroded Pribnow box. Mutations rendering the Pribnow promoter box more similar to the prokaryotic consensus sequence significantly increased expression, even within lysogens (cf. constitutively derepressed Pid/*dgo* interaction), and even in the absence of any other P22-related factors (cf. reporter plasmid studies). This in turn suggests that the Pid overexpression observed during the phage carrier state of P22wt [11] could stem from P22 copy-number (i.e., *pid* dosage) effects during episome formation, rather than from more complex regulatory effects.

The *pid* promoter, however, was found to be located far upstream of the start codon, and TSS analysis confirmed the presence of an extensive 174 bp 5′UTR. While long 5′UTR regions have been linked to mRNA stability [23,24,25] and expression regulation [26] in bacteria and archaea, the prevalence of long 5′UTRs in phages remains relatively uncommon and unexplored. The filamentous phage SW1, isolated from the deep-sea bacterium *Shewanella piezotolerans* WP3, contains two operons that have exceptionally long 5′UTRs (with lengths of 314 and 601 bp) that appear to thermoregulate the RNA stability of the transcripts [27]. Our results suggest that the 5′UTR of *pid* is responsible for the appearance of a second (and smaller) *pid* mRNA species, which could originate from an RNA processing site or the presence of a yet undetected secondary promoter. Although it currently remains to be resolved whether this smaller species serves a regulatory function, previous research has shown that mRNA processing in the 5′UTR region can affect transcript stability [28,29,30]. 

The 3′UTR of *pid* contains a functional Rho-independent termination site, as suggested by the mutation attenuating the Pid/*dgo* interaction and *pid* transcript instability. Previous studies suggest that disruption of the stem-loop structure of Rho-independent termination sites abolishes terminator activity [13,31,32]. As a consequence, the mRNA is degraded and expression of the ORF is drastically decreased. This is exemplified by the *tI* terminator of phage *λ*, in which disruption of the stem structure via the introduction of point mutations or deletions significantly reduces termination efficiency and decreases RNA stability [31,32]. Similarly, in *E. coli*, the disruption of a G+C-rich stem structure caused almost complete abolition of terminator activity and reduced expression of the *crp* gene due to increased mRNA degradation [13].

In summary, the expression regulation mechanism of the P22 *pid* ORFan gene was scrutinized, revealing a weak promoter region with an extensive 5′UTR region. The latter may harbor a transcript processing or secondary promoter site, although its biological significance still remains elusive. Termination of the *pid* transcript is controlled by a Rho-independent terminator that is important for proper *pid* expression. As such, this study provides functional characterization of one of the few remaining ORFans in the P22 model phage.

## 4. Materials and Methods

### 4.1. Strains, Phages, and Growth Conditions 

Bacterial strains, phages, and plasmids used in this study are listed in Table 1. Lysogeny broth (LB) medium [33] was used to culture bacteria, either as a broth or as agar plates after the addition of 1.5% (for spreading plates) or 0.7% (for soft agar plates) agar. Stationary phase cultures were obtained by growing cultures in LB for 15–20 h at 37 °C under aerated conditions (agitation of 200 rpm on rotary shaker). Exponential phase cultures were obtained by 1:100 dilution of stationary phase cultures in pre-warmed broth and further grown at 37 °C to an OD_600_ between 0.2 and 0.3. When required, the following chemicals (AppliChem GmbH, Darmstadt, Germany) were added to the growth medium at the indicated final concentrations: kanamycin (50 µg/mL), ampicillin (100 µg/mL), tetracycline (20 µg/mL), and 5-bromo-4-chloro-3-indolyl-β-D-galactopyranoside (40 µg/mL; X-Gal).

Phages were propagated on *S.* Typhimurium LT2 via the double agar overlay method in LB soft agar [34]. Three plaques were subsequently resuspended in 1 mL phage buffer (10 mM Tris-HCl (pH 7.5), 10 mM MgSO_4_, 150 mM NaCl) and incubated at 37 °C, 1 h, agitation of 200 rpm on rotary shaker. A dilution series of the supernatant was plated with 100 µL of stationary phase LT2 in soft agar (double agar overlay method) and incubated at 37 °C for 15–20 h. The soft agar layer was scraped off, chloroform was added, and the mixture was centrifuged for 10 min (6000 rpm). The supernatant was filter sterilized with 0.2 µm PVDF membrane filters (Fisher Scientific, Hampton, NH, USA), and chloroform was added. 

### 4.2. Construction of Lysogens

Lysogens were constructed by propagation of a phage on indicator strain LT2K7 via the double agar overlay method [34]. Cells within the turbid zone of a plaque were isolated and purified on green indicator plates [39] and plates were incubated for 15–20 h at 37 °C. Pale green colonies were selected and cross streaked across the respective phage on green indicator plates to confirm lysogenisation. 

### 4.3. Construction of Phage Mutants

Amplicons used in the construction of phage mutants were obtained via PCR with Phusion DNA polymerase (Thermo Fisher Scientific, Waltham, MA, USA). Primers were synthesized by IDT (Leuven, Belgium) and are listed in Table 2.

P22-Δ*pid* was constructed on the basis of the *λ-red* recombineering system, described by Datsenko and Wanner in the year 2000 [37]. The amplicon was generated via PCR amplification with pKD13 as template and primer pair P22_Δpid_Fw and P22_Δpid_Rev. The introduced kanamycin cassette was subsequently flipped out with pCP20, which harbors Flp to recombine two *frt*-sites flanking the kanamycin gene [38]. 

Phage mutants P22-*pid*^423^, P22-*pid*^371^, and P22-*pid*^331^ were constructed on the basis of the *λ-red* recombineering system [37] and a two-step selection and counterselection process using the *tetA-sacB* cassette, described by Li et al. in the year 2013 [36]. The *tetA-sacB* cassette was amplified from *E. coli* XTL298 in a colony PCR with primers P22_5UTRpid_tetAsacB_Fw and P22_5UTRpid_tetAsacB_Rev. The obtained cassette was introduced in the 5′UTR region of *pid* via homologous recombination, replacing 13–157 bp upstream of the *pid* ORF. Correct clones were selected on tetracycline LB agar plates. The *tetA-sacB* cassette was subsequently substituted via homologous recombination with the truncated 5′UTR amplicons obtained via either primer pairs P22_5UTR423pid_Fw and P225UTRpid_Rev, P22_5UTR371pid_Fw and P225UTRpid_Rev, or P22_5UTR331pid_Fw and P225UTRpid_Rev. Correct clones were selected on counterselection agar plates and validated via Sanger sequencing (Macrogen Europe B.V., Amsterdam, the Netherlands). 

### 4.4. Construction of Plasmids

The inserts that were ligated in the vectors were obtained via PCR with Phusion DNA polymerase (Thermo Fisher Scientific, Waltham, MA, USA). Primers were synthesized by IDT (Leuven, Belgium) and can be found in Table 2. Constructs were validated via Sanger sequencing (Macrogen Europe B.V., Amsterdam, the Netherlands).

Plasmids pFPV-P22-P*pid^wt^-gfp*, pFPV-P22-P*pid^C–182A^-gfp*, and pFPV-P22-P*pid^C–183A^-gfp* were constructed by digesting pFPV25 with XbaI and BamHI (Thermo Fisher Scientific, Waltham, MA, USA). The digested vector was subsequently ligated with the (mutated) 5′ regulatory region of *pid*. The latter amplicon was obtained using primers P22_pid_Fw and P22_pid_Rev and digested with XbaI and BamHI, prior to ligation. 

Plasmids pFPV-P22-P*pid*^338^*-gfp* and pFPV-P22-P*pid*^232^*-gfp* were constructed by digesting pFPV25 with XbaI and BamHI (Thermo Fisher Scientific, Waltham, MA, USA). The digested vector was subsequently ligated with a truncated 5′ regulatory region of *pid* of either 338 bp or 232 bp. The latter amplicons were obtained, respectively, using primer pairs P22_pid338_Fw and P22_pid_Rev or P22_pid232_Fw and P22_pid_Rev, and digested with XbaI and BamHI prior to ligation. 

### 4.5. DES-Mutagenesis Screen

To screen for mutants in P22, we added 50 µL of the mutagen diethylsulfate (DES) to 50 µL of LT2 [P22] stationary phase culture and the mixture was incubated for 50 min at 37 °C without shaking. Next, 50 µL of the mixture was added to 2 mL of LB broth and grown for 6 h at 37 °C under shaking conditions. Phages were extracted and propagated on LT2 as plaques in LB soft agar or as lysates in LB. This procedure was repeated eight times to obtain eight pools of mutagenized phage lysate. The mutagenized phage lysate was propagated on stationary phase culture of LT2K7 on green indicator plates [39]. After overnight incubation, each indicator plate was replica plated on a new indicator plate and on a LB agar plate containing X-Gal. In total, 39,950 plaques were screened on LB agar plates supplemented with 40 µg/mL X-Gal and 26,740 plaques on LB agar plates supplemented with 20 µg/mL X-Gal, allowing the identification of more blue phenotypes compared to wild-type P22.

### 4.6. β-Galactosidase Assay

Expression of β-galactosidase (LacZ) was visualized by adding 5-bromo-4-chloro-3-indolyl-β-D-galactopyranoside (X-Gal) to agar plates (40 µg/mL). β-Galactosidase hydrolyses X-Gal to an insoluble blue precipitate. For clear visualization of spotted phage populations, we pressed agar plates onto Whatman filter papers (GE Healthcare, Chicago, IL, USA) before photographing them. 

### 4.7. Fluorescence Population Level Measurement

Population-level fluorescence measurements were obtained in a Fluoroskan Ascent FL (Thermo Fisher Scientific, Waltham, MA, USA), equipped with a filter pair with an excitation wavelength of 485 nm and an emission wavelength of 520 nm. A multi-well microtiter plate was filled with 200 µL of stationary phase cultures grown in AB-glucose medium (2 g/L (NH_4_)_2_SO_4_, 6 g/L Na_2_HPO_4_, 3 g/L KH_2_PO_4_, 3 g/L NaCl, 0.1 mM CaCl_2_, 1.0 mM MgCl_2_, and 0.003 mM FeCl_3_ [40] and supplemented with 0.2% D-glucose, 1 μg/mL uracil, and 1 μg/mL thiamine and 0.2% casamino acids). The obtained fluorescence values were subsequently normalized over the optical density at OD_600_ of the sample (obtained via the Multiskan RC, Thermo Labsystems OY, Helsinki, Finland).

### 4.8. Statistical Analysis

All fluorescence measurements were performed in triplicate. Statistical analyses, histograms, and Tukey HSD tests (*p* = 0.05) were carried out using the JMP software (version Pro 15.1.0; SAS, Cary, NC, USA), and differences were considered significant when *p* ≤ 0.05.

### 4.9. Northern Blotting 

Exponential phase cultures of *S.* Typhimurium LT2 were infected with P22wt, P22-*pid*^423^, P22-*pid*^371^, P22-*pid*^331^, P22-*pid^C–183A^*, P22-*pid^term_G292A^*, or P22-Δ*pid* (MOI 10) for 90 min. Infection was stopped by adding 20% volume of stop mixture (95% EtOH, 5% phenol; pH 5.5), followed by snap-freezing in liquid nitrogen. Following this, total RNA was extracted according to the hot phenol method, and the remaining DNA was digested via DNase I treatment (Thermo Fisher Scientific, Waltham, MA, USA). Subsequently, 7 µg of total RNA per sample was resolved on a 6% polyacrylamide gel containing 7 M urea and transferred to an Amersham Hybond-XL (GE Healthcare, Chicago, IL, USA) membrane. After UV-crosslinking, transcripts of interest were detected by radiolabeled oligonucleotides Sal_5S_rRNA (5S rRNA probe for loading control), Pid_5UTR, and/or Pid_ORF (Table 2). The labelling reaction was carried out as follows: 10 pmol of the oligonucleotides were 5′-labeled with 10 µCi ^32^P-γ-ATP (Perkin Elmer, Waltham, MA, USA) by T4 polynucleotide kinase (PNK, Thermo Fisher Scientific, Waltham, MA, USA) for 1 h at 37 °C. After removing unincorporated ^32^P-γ-ATP with a Microspin G-25 column (GE Healthcare, Chicago, IL, USA), 7 µL of the labelled oligonucleotide was added to 17 mL EKONO hybridization buffer (G-Biosciences, Saint Louis, MI, USA) and hybridized to the membrane, overnight at 42 °C in a hybridization oven. Radioactive signals were imaged with a Typhoon 9400 (Variable Mode Imager, Amersham Biosciences, Amersham, United Kingdom), and the membrane was stripped for re-probing by immersing it in 500 mL hot H_2_O/5 mL 10% SDS for 7 min.

## Figures and Tables

**Table 1:** Strains, bacteriophages, and plasmids used in this study.

Name	Characteristic	Source or Reference
Strains		
LT2	*Salmonella* Typhimurium wild-type	[35]
LT2K7	LT2 *dgoT::*Mu*d*K$Insertion of Mu*d*K in the *dgo* operon, resulting in a translational *lacZ* reporter fusion to the *dgoT* gene	[12]
LT2 [P22wt]	LT2 lysogenized with P22wt	This study
LT2K7 [P22wt]	LT2K7 lysogenized with P22wt	This study
LT2K7 [P22-Δ*pid*]	LT2K7 lysogenized with P22-Δ*pid*	This study
LT2K7 [P22-*pid^C–183A^*]	LT2K7 lysogenized with P22-*pid^C–183A^*	This study
LT2K7 [P22-*pid^C–182A^*]	LT2K7 lysogenized with P22-*pid^C–182A^*	This study
D23580	*Salmonella* Typhimurium strain, naturally lysogenized with BTP1 prophage	[20]
*E. coli* XTL298	Contains the *tetA-sacB* cassette	[36]
Bacteriophages		
P22wt	Wild-type P22 phage	Salmonella Genetic Stock Centre (SGSC) ^1^
P22-Δ*pid*	P22 with a deletion of the *pid* ORF and regulatory region	This study
P22-*pid^ORF_C251T^*	P22 with a C-to-T point mutation, 251 bp downstream of the *pid* start codon	This study
P22-*pid^term_G292A^*	P22 with a G-to-A point mutation in the rho-independent terminator, 292 bp downstream of the *pid* start codon	This study
P22-*pid^C–183A^*	P22 with a C-to-A point mutation, 183 bp upstream of the *pid* start codon	This study
P22-*pid^C–182A^*	P22 with a C-to-A point mutation, 182 bp upstream of the *pid* start codon	This study
P22-*pid*^423^	P22 with a 3′ truncated 5′UTR region of 109 bp, followed by an intact *pid* RBS	This study
P22-*pid*^371^	P22 with a 3′ truncated 5′UTR region of 57 bp, followed by an intact *pid* RBS	This study
P22-*pid*^331^	P22 with a 3′ truncated 5′UTR region of 17 bp, followed by an intact *pid* RBS	This study
Plasmids		
pKD46	Encodes *λ-red* recombineering genes under control of L-arabinose inducible promoter	[37]
pKD13	Harbors *frt-kan-frt* site for construction of deletions by recombineering	[37]
pCP20	Encodes flippase (FLP) for recombining *frt* sites	[38]
pFPV25	Encodes promoterless GFP	[17]
pFPV-P22-P*pid^wt^-gfp*	Contains 521 bp of the 5′ regulatory region of P22wt upstream of *gfp* in the pFPV25 plasmid	This study
pFPV-P22-P*pid^C–182A^-gfp*	Contains 521 bp of the 5′ regulatory region of P22-P*pid^C–182A^* upstream of *gfp* in the pFPV25 plasmid	This study
pFPV-P22-P*pid^C–183A^-gfp*	Contains 521 bp of the 5′ regulatory region of P22-P*pid^C–183A^* upstream of *gfp* in the pFPV25 plasmid	This study
pFPV-P22-P*pid*^338^*-gfp*	Contains 338 bp of the 5′ regulatory region of P22wt upstream of *gfp* in the pFPV25 plasmid	This study
pFPV-P22-P*pid*^232^*-gfp*	Contains 232 bp of the 5′ regulatory region of P22wt upstream of *gfp* in the pFPV25 plasmid	This study

^1^ https://people.ucalgary.ca/~kesander/ (accessed on 27 December 2021).

**Table 2:** Primers used in the study.

Primer Name	Sequence (5′–3′) ^1^
P22_Δpid_Fw	GTGATGATGCCGAGCACGCCCATCTGGACTATCTCAACTAGTCGATTCAT **ATTCCGGGGATCCGTCGACC**
P22_Δpid_Rev	CTTATACCATCGACTGGATATTATTCGTTTTATCCCGTCTATGTGGGGGGGGGGATAAAA **TGTAGGCTGGAGCTGCTTCG**
P22_pid_Fw	GTCA*GGATCC* **ACAGGTCTAACGCTTCCC**
P22_pid_Rev	GATG*TCTAGA* **GCATAAAGTTTCTTGTGGTTG**
P22_pid338_Fw	GTCA*GGATCC* **ACTGGAATTTCTGTTCTTCAGTCA**
P22_pid232_Fw	GTCA*GGATCC* **TCATGACATGTGTCACATTTATA**
P22_5UTRpid_tetAsacB_Fw	ATATCTTCAAGGTGGGCAATTTTTTGCTCTATATCTGACATGTCCACTCCTTT **TCCTAATTTTTGTTGACACTCTATC**
P22_5UTRpid_tetAsacB_Revv	ATTGCTTTAAGTTTACAGAACAATAATCCTTGGCTGGACGTAAGGTTTTGACA **ATCAAAGGGAAAACTGTCCATATGC**
P22_5UTR423pid_Fw	ATATCTTCAAGGTGGGCAATTTTTTGCTCTATATCTGACATGTCCACTCCTTT **TCTGTCATGAGTACCTCATCG**
P22_5UTR371pid_Fw	ATATCTTCAAGGTGGGCAATTTTTTGCTCTATATCTGACATGTCCACTCCTTT **GGAGATATCTAAGATTGC**
P22_5UTR331pid_Fw	ATATCTTCAAGGTGGGCAATTTTTTGCTCTATATCTGACATGTCCACTCCTTT **TGTCAAAACCTTACGTCCAGCCAA**
P225UTRpid_Rev	**GATTCATGACATGTGTCACATTTATACCAACCAGATCATTGCTTTAAGTTTAC**
Sal_5S_rRNA	**TGGGACCACCGCGCTAGTGCCG**
Pid_5UTR	**ATCTAAGATTGCTATCACACTGC**
Pid_ORF	**GATAATATCTTCAAGGTGGGCA**

^1^ Primer attachment sites are indicated in bold. Restriction sites are shown in italics. Recombination regions are indicated in regular font.

## Data Availability

The data presented in this study are available on request from the corresponding author.
